# Cutaneous Ulcers and Response to Treatment in a Child with Anti-MDA5 Dermatomyositis

**DOI:** 10.31138/mjr.191123.cua

**Published:** 2024-05-21

**Authors:** Mamadapur Mahabaleshwar, Ramaswamy Subramanian

**Affiliations:** Clinical Immunology and Rheumatology, JSS Medical College, JSS Medical Institutions, Mysore, India

**Keywords:** anti-MDA5, juvenile dermatomyositis, ulcer

## CLINICAL IMAGE

**Figure 1.**
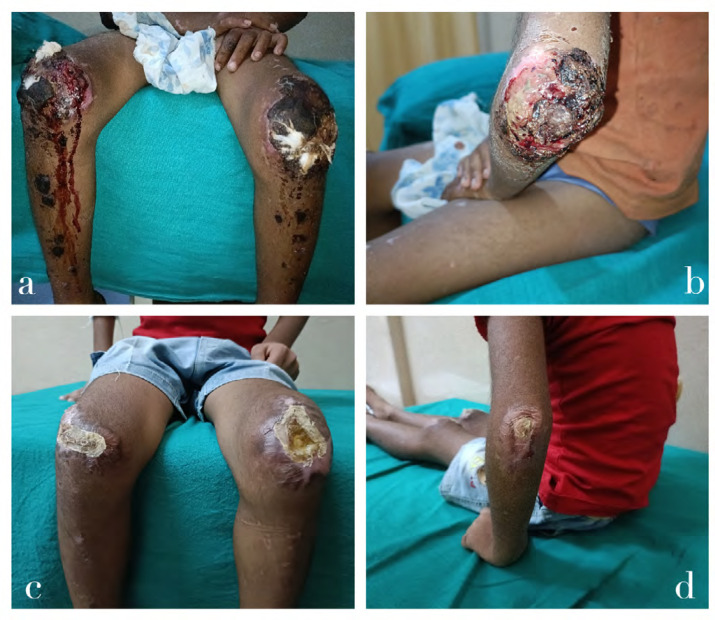
**(A–B)** Ulcers over both knees and elbow with crusting. **(C–D)** Healed ulcers at follow-up following immuno-suppression.

A 6-year-old female presented with painful skin ulcers with discharge over both elbows and knees for 10 days. There was no fever, muscle weakness, oral ulcers, joint pain, or cardiorespiratory complaints. On examination, she had ulcers over both elbows, knees, and lateral malleoli with crusting. Gottron papules were present (**Figure 1A–B**). Muscle power was 5/5. Investigation revealed CRP of 48.61 mg/L, Hb of 9.4gm/dl. CPK was 25U/L. Serum LDH was 251 IU/L.LFT was normal. ANA was negative. Her extended myositis profile was positive for Anti-MDA5 antibody. CT chest was normal. She was managed with IV immunoglobulin 2gm/kg over 5 days, and IV methylprednisolone 15mg/kg for 3 days. She was discharged with Prednisolone and Tacrolimus. One month later the skin ulcers had healed (**Figure 1C–D**).

## DISCUSSION

Juvenile Dermatomyositis is an autoimmune disease characterised by inflammation, autoimmunity, and vasculopathy^1^ with protean manifestations. Juvenile MDA 5 myositis prevalence may vary from 10–40% of JDM cases.^2^ Antimelanoma differentiation-associated gene 5 (MDA5) antibody-positive dermatomyositis usually presents with minimal or no muscle weakness,^3,4^ cutaneous ulcers which may be deep and punched out in the extensor aspects, arthritis, and ILD. Treatment recommendations vary in the management of anti-MDA5 JDM. The triple combination therapy (high-dose glucocorticoids, calcineurin inhibitor, and intravenous cyclophosphamide) has been widely used. Evidence for the role of JAK inhibitors, rituximab, plasma exchange, and polymyxin B perfusion is limited and is used in refractory cases.^5^

Early diagnosis and initiation of aggressive combined immunosuppression help reduce damage and improve survival.
